# Bat Nipah Virus, Thailand

**DOI:** 10.3201/eid1112.050613

**Published:** 2005-12

**Authors:** Supaporn Wacharapluesadee, Boonlert Lumlertdacha, Kalyanee Boongird, Sawai Wanghongsa, Lawan Chanhome, Pierre Rollin, Patrick Stockton, Charles E. Rupprecht, Thomas G. Ksiazek, Thiravat Hemachudha

**Affiliations:** * Chulalongkorn University Hospital, Bangkok, Thailand; † Thai Red Cross Society, Bangkok, Thailand; ‡ Ministry of Natural Resources and Environment, Bangkok, Thailand; § Centers for Disease Control and Prevention, Atlanta, Georgia, USA

**Keywords:** Nipah virus, Hendra virus, RNA virus, bat, chiroptera, zoonosis, animals, Thailand, serology, dispatch

## Abstract

Surveillance for Nipah virus (NV) was conducted in Thailand's bat population. Immunoglobulin G antibodies to NV were detected with enzyme immunoassay in 82 of 1,304 bats. NV RNA was found in bat saliva and urine. These data suggest the persistence of NV infection in Thai bats.

Nipah virus (NV) caused a major outbreak in swine and humans in Malaysia from September 1998 to April 1999 that led to 265 human cases with 105 deaths and the culling of >1 million swine (*1*). The genesis of the outbreak was suggested to be associated with bats (*2**,**3*). NV and Hendra virus (HV) are members of the *Paramyxoviridae* family in the genus *Henipavirus* (*4*). A seroepidemiologic study in Malaysia implicated 4 fruit bat species, *Pteropus hypomelanus*, *P. vampyrus*, *Cynopterus brachyotis*, *Eonycteris spelaea*, and an insectivorous bat, *Scotophilus kuhlii* (*2*). NV was also identified and isolated from bat urine samples of *P. hypomelanus* (*5*). Unlike NV's first appearance in Malaysia, in outbreaks in Bangladesh, infection may have been contracted by eating fruits contaminated with bat saliva, and transmitted from person to person (*6*). Antibodies to NV antigen were detected in 2 *P. giganteus* adult females from Bangladesh (*6*). Recently, antibodies to NV and virus isolation were successfully demonstrated in *P. lylei* from Cambodia (*7*).

Thailand is bordered by Malaysia to the south and Cambodia to the southeast. No NV infections in humans have been reported in Thailand. Surveillance in swine by enzyme-linked immunosorbent assay (ELISA) showed negative results (*8*). Estimates suggest ≈112 bat species in Thailand; 18 are fruit bats and 94 are insectivorous bats (*9*). Given that NV has caused several outbreaks in the region, obtaining baseline data for surveillance and planning for future public health assessment of its impact are essential.

## The Study

From March 2002 to February 2004, a total of 17 trips were made to 15 sites in 9 provinces in central, eastern, and southern Thailand (Figure). Bats were caught and blood samples were collected as previously described (*10*). Of 12 bat species collected, 6 were frugivorous and 6 were insectivorous (Figure). Seventy-one percent (932) of 1,304 samples were from *Pteropus* bats and 66% (857) were from *P. lylei*. Saliva and urine were obtained by swabbing and stored in tubes with 1.0 mL of NucliSens lysis buffer containing guanidine thiocyanate (bioMérieux, Boxtel, the Netherlands) for transporting. Liquid from ≈10 individual samples from the same species, colony, and time of capture was saved into the same pool. A total of 142 pools each were collected from 1,286 saliva and 1,282 urine specimens. The pooled specimens were frozen at –70°C until analysis.

**Figure:**
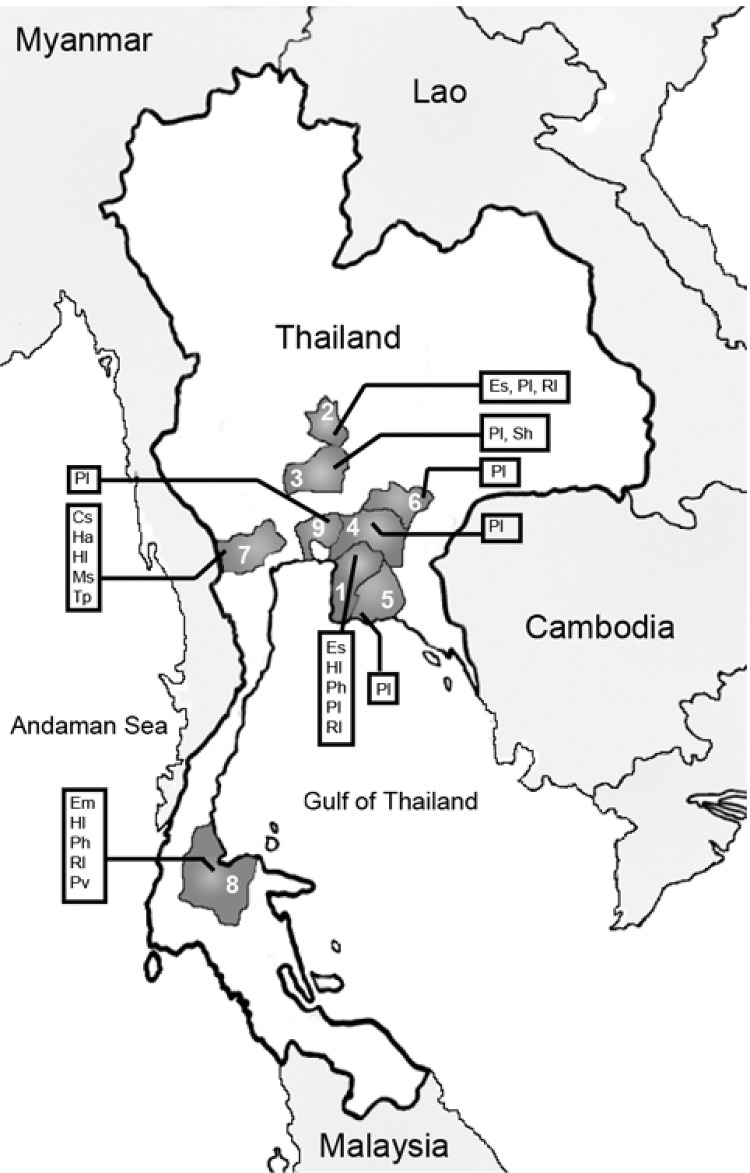
Locations in Thailand where bats have been captured. 1 = Chon Buri, 2 = Sing Buri, 3 = Ayutthaya, 4 = Cha Choeng Sao, 5 = Ra Yong, 6 = Pra Chin Buri, 7 = Ratcha Buri, 8 = Surat Thani, 9 = Bangkok. Species analyzed: Cs = Cynopterus sphinx, Em=Emballonura monticola, Es = Eonycteris spelaea, Ha = Hipposideros armiger, Hl = Hipposideros larvatus, Ms = Megaderma spasma, Ph = Pteropus hypomelanus, Pl = P. lylei, Pv = P. vampyrus, Rs = Rousettus leschenaulti, Sh = Scotophilus heathi, Tp = Tadarida plicata.

Immunoglobulin G (IgG) antibodies to NV were assayed by indirect ELISA at Chulalongkorn University Hospital, with a protocol developed by the Centers for Disease Control and Prevention (CDC), Atlanta, Georgia. Serum samples were heated to 56°C and titrated at 4 dilutions (1:100, 1:400, 1:1,600, and 1:6,400). Of the 1,054 serum specimens tested, 82 (7.8%) from 4 species—*P. hypomelanus* (n = 4), *P. lylei*, (n = 76), *P. vampyrus* (n = 1), and *Hipposideros larvatus* (n = 1)—were NV IgG antibody–positive (titer >1:400) with 43 at a titer of 1:400; 30 at 1:1,600, and 9 at 1:6,400. *P. lylei* contained higher serum antibody titers than other species (9 of 76 at 1:6,400, 29 of 76 at 1:1,600) (Table).

**Table:** ELISA, PCR saliva, and PCR urine results for Nipah virus from 12 bat species, Thailand, 2002–2004*

Species		Total bats	ELISA		PCR saliva‡		PCR urine‡	
			No. analyzed	No. positive (%) †	No. analyzed	No. pool positive/total	No. analyzed	No. pool positive/total
Frugivorous								
	*Cynopterus sphinx*	34	10	0	34	0/5	34	0/5
	*Eonycteris spelaea*	64	54	0	64	0/7	64	0/7
	*Pteropus hypomelanus*	36	26	4 (15.4)	36	0/6	35	0/6
	*P*. *lylei*	857	813	76 (9.3)	845	1/87	845	6/87
	*P*. *vampyrus*	39	39	1 (2.6)	39	0/4	39	0/4
	*Rousettus leschenaulti*	11	4	0	6	0/3	6	0/3
Insectivorous								
	*Emballonura monticola*	14	12	0	14	0/2	14	0/2
	*Hipposideros armiger*	88	6	0	88	0/10	88	0/10
	*H*. *larvatus*	95	74	1 (1.3)	94	1/10	91	0/10
	*Megaderma spasma*	13	0	0	13	0/2	13	0/2
	*Scotophilus heathi*	3	3	0	3	0/1	3	0/1
	*Tadarida plicata*	50	13	0	50	0/5	50	0/5
Total		1,304	1,054	82 (7.8)	1,286	2/142	1,282	6/142

*ELISA, enzyme-linked immunosorbent assay; PCR, polymerase chain reaction.

†ELISA positive: titer >1:400.

‡10 individual samples (saliva or urine) from the same species, colony, and the time of capture were saved into the same pool.

Total RNA was extracted from saliva and urine according to manufacturer's protocol. A RNA plasmid was introduced as an internal control RNA in the duplex reverse transcription–polymerase chain reaction (RT-PCR) as previously described (*11*). NV nucleoprotein (N)-specific primers used for reverse transcription and first-round PCR were: NP1F, 5´ CTT GAG CCT ATG TAT TTC AGA C 3´; NP1R, 5´ GCT TTT GCA GCC AGT CTT G 3´. The internal primers for nested PCR were previously described (*1*). This process allowed an internal control to be visualized as the upper (323 bp) bands and NV product as lower bands (227 bp). Single-step RT-PCR was performed by using the One Step RT-PCR kit (Qiagen Inc., Valencia, CA, USA) followed by nested PCR. The PCR product was sized by gel electrophoresis in 2% agarose. Only samples showing both the 323-bp internal control and 227-bp NV-specific bands, or only a NV-specific band, were considered positive; those showing only the internal control band were considered negative. Those showing no band were tested again and judged to contain enzyme inhibitors if no band was shown on repetition. All samples with positive results were tested again without the positive control, and the sequence of amplified product was determined by using internal primer.

The sensitivity of the duplex system is not notably altered by incorporation of the internal control RNA (data not shown). Samples from a saliva pool of *H. larvatu*s from site 1 in Chon Buri Province and another pool of *P. lylei* from site 3 in Chon Buri Province were duplex nRT-PCR positive. All 6 positive duplex nRT-PCR urine pools were collected from *P. lylei* captured from 3 different sites, 1 from Cha Choeng Sao, 1 from Bangkok, and 4 from site 3 in Chon Buri. The 181-nucleotide (nt) sequences of the N gene obtained from 1 saliva pool of *H. larvatu*s was identical to those reported from Malaysia (accession no. NC_002728). The sequences of 1 saliva pool from *P. lylei* and 6 urine pools from *P. lylei* were identical to those reported from Bangladesh (AY988601) with 13 divergent nt (92% identity) from Malaysia. The nucleotide changes at positions 1397, 1407, and 1481 resulted in amino acid substitutions (with 94% identity to Malaysia, 56 of 59) from isoleucine to valine, glycine to glutamic acid, and asparagine to aspartic acid at codons 429, 432, and 457 of N protein, respectively. Nine divergent nucleotides among Thai, Bangladesh, and Cambodia (AY858110) did not result in amino acid differences.

## Conclusions

This study reports the evidence of NV infection in Thai frugivorous and insectivorous bats demonstrated by IgG antibodies to NV in serum samples and NV RNA in urine and saliva. Antibodies against NV were detected in *P. hypomelanus*, *P. vampyrus*, *P. lylei*, and *H. larvatus*. NV infections in the first 2 species were similar to those reported in Malaysia (*2*). *P. lylei* was the only bat species found NV-infected among 14 species tested in Cambodia (*7*). An earlier report demonstrated a correlation between ELISA and neutralization tests with 87% sensitivity and 99% specificity (*7*). These data support our ELISA results as a firstline screening tool to investigate NV infection in countries that do not have a BSL-4 facility in which to perform neutralization assays. The finding of unusually high antibody titers from *P. lylei* suggests that NV circulates mainly in this bat species in Thailand and Cambodia (*7*).

Although serum neutralization tests were not conducted, NV RNA was demonstrated in saliva and urine from *P. lylei* and saliva of *H. larvatus*. Determining PCR positivity by naked eye observations for the presence of a 227-bp fragment is not likely the most sensitive method (our detection limit is 0.37 pg total RNA/μL); therefore, some low-positive samples might be missed. Increasing the volume of sample tested by using a plastic sheet method in urine collection may overcome such problems (*12*).

Southern blot analysis is also useful for PCR confirmation; however, sensitivity may not be markedly improved as previously reported in the case of rabies (*13*). We used a nested PCR method because less RNA was required initially and because of a shorter turnaround time. Confirmation was achieved by direct sequencing of amplified products. Taken together, our current ELISA and PCR data are sufficient to conclude that Thai bats were naturally infected with NV. Higher numbers of PCR-positive samples in *P. lylei* may be due to a bias in species collection. Alternatively, in the serologic study, *P. lylei* may be the most prevalent infected species. Sequence analysis of the short 181-nt sequence suggests that >2 strains of NV are circulating in Thai bats. More sequence data are required to confirm this hypothesis. Finding NV RNA in saliva of *H. larvatus*, may indicate the insectivorous bat as another reservoir or this may be only an accidental spillage.

We believe that NV infection is prevalent in Thai fruit bats as previously reported in Malaysia and Cambodia (*2**,**7*). Countrywide surveillance is needed to clarify the epidemiology of NV infection in Thailand as it relates to host, seasonal, and geographic attributes.

## References

[1] Chua KB, Bellini WJ, Rota PA, Harcourt BH, Tamin A, Lam SK, Nipah virus: a recently emergent deadly paramyxovirus. Science. 2000;288:1432–5. 10.1126/science.288.5470.1432 10827955

[2] Yob JM, Field H, Rashdi AM, Morrissy C, van der Heide B, Rota P, Nipah virus infection in bats (order *Chiroptera*) in peninsular Malaysia. Emerg Infect Dis. 2001;7:439–41. 11384522 10.3201/eid0703.010312 PMC2631791

[3] AbuBakar S, Chang LY, Ali AR, Sharifah SH, Yusoff K, Zamrod Z. Isolation and molecular identification of Nipah virus from pigs. Emerg Infect Dis. 2004;10:2228–30. 15663869 10.3201/eid1012.040452 PMC3323361

[4] Wang LF, Yu M, Hansson E, Pritchard LI, Shiell B, Michalaki WP, The exceptionally large genome of Hendra virus: support for creation of a new genus within the family *Paramyxoviridae.* J Virol. 2000;74:9972–9. 10.1128/JVI.74.21.9972-9979.2000 11024125 PMC102035

[5] Chua KB, Koh CL, Hooi PS, Wee KF, Khong JH, Chua BH, Isolation of Nipah virus from Malaysian Island flying-foxes. Microbes Infect. 2002;4:145–51. 10.1016/S1286-4579(01)01522-2 11880045

[6] Hsu VP, Hossain MJ, Parashar UD, Ali MM, Ksiazek TG, Kuzmin I, Nipah virus encephalitis reemergence, Bangladesh. Emerg Infect Dis. 2004;10:2082–7. 15663842 10.3201/eid1012.040701 PMC3323384

[7] Reynes J-M, Counor D, Ong S, Faure C, Seng V, Molia S, Nipah virus in Lyle's flying foxes, Cambodia. Emerg Infect Dis. 2005;11:1042–7. 16022778 10.3201/eid1107.041350 PMC3371782

[8] Damrongwatanapokin S. Situation and surveillance of Nipah virus infection of pigs in Thailand. In: Proceedings of the challenge of infectious diseases in the 21st century. Chiangmai, Thailand; 2005 Jun 15–17. Bangkok, Thailand: Bureau of General Communicable Diseases, Department of Disease Control; 2005. p. 172–87.

[9] Boonkird K, Wanghongsa S. Diversity of bats in Thailand. In: Compilation of 2003 research, progressive reports and essays on wildlife ecology. Bangkok, Thailand: Wildlife Research Division, Department of National Park, Wildlife and Plant Conservation; 2004. p. 183–96.

[10] Lumlertdaecha B, Boongird K, Wanghongsa S, Wacharapluesadee S, Chanhome L, Khawplod P, Survey for bat lyssaviruses, Thailand. Emerg Infect Dis. 2005;11:232–6. 15752440 10.3201/eid1102.040691 PMC3320458

[11] Echevarria JE, Avellon A, Juste J, Vera M, Ibanez C. Screening of active lyssavirus infection in wild bat populations by viral RNA detection on oropharyngeal swabs. J Clin Microbiol. 2001;39:3678–83. 10.1128/JCM.39.10.3678-3683.2001 11574590 PMC88406

[12] Chua KB. A novel approach for collecting samples from fruit bats for isolation of infectious agents. Microbes Infect. 2003;5:487–90. 10.1016/S1286-4579(03)00067-4 12758277

[13] Smith J, McElhinney LM, Heaton PR, Black EM, Lowings JP. Assessment of template quality by the incorporation of an internal control into a RT-PCR for the detection of rabies and rabies-related viruses. J Virol Methods. 2000;84:107–15. 10.1016/S0166-0934(99)00124-X 10680960

